# Effectiveness of Erythritol-Based Air Polishing and Ultrasonic Instrumentation with PEEK Inserts in Peri-Implant Maintenance: A Randomized Clinical Trial Including Different Prosthetic Materials

**DOI:** 10.3390/dj13060235

**Published:** 2025-05-26

**Authors:** Carolina Maiorani, Andrea Butera, Carlos Pérez-Albacete Martínez, Maurizio Pascadopoli, Silvia Sabatini, Gianna Maria Nardi, Andrea Scribante

**Affiliations:** 1 Unit of Dental Hygiene, Section of Dentistry, Department of Clinical, Surgical, Diagnostic and Pediatric Sciences, University of Pavia, 27100 Pavia, Italy; carolina.maiorani@unipv.it (C.M.); andrea.scribante@unipv.it (A.S.); 2 Health Sciences PhD Programme, Universidad Catòlica de Murcia UCAM, Campus de Ios Jerònimos N. 135, 30107 Guadalupe, Murcia, Spain; 3 Tissue Regeneration and Repair Group, Biomaterials and Tissue Engineering, Faculty of Health Sciences, UCAM—Universidad Catòlica San Antonio de Murcia, 30107 Guadalupe, Murcia, Spain; cperezalbacete@ucam.edu; 4 Unit of Orthodontics and Pediatric Dentistry, Section of Dentistry, Department of Clinical, Surgical, Diagnostic and Pediatric Sciences, University of Pavia, 27100 Pavia, Italy; 5 Department of Surgical, Medical, Dental and Morphological Sciences, University of Modena and Reggio Emilia, 41124 Modena, Italy; silviasabatini01@gmail.com; 6 Department of Odontostomatological and Maxillofacial Sciences, Sapienza University of Rome, 00161 Rome, Italy; giannamaria.nardi@uniroma1.it

**Keywords:** dental implant, peri-implant diseases, erythritol, ultrasonic

## Abstract

Background: Peri-implant diseases, including mucositis and peri-implantitis, pose a challenge to implant dentistry and require effective maintenance protocols. Professional biofilm removal is essential for peri-implant health, but the optimal decontamination method remains controversial. Methods: This randomized clinical trial compared erythritol-based air polishing and ultrasonic instruments with PEEK (polyetheretherketone) inserts in peri-implant maintenance, also regarding the different prosthetic materials. A total of 120 patients with implant-supported feldspar ceramic, zirconia, or lithium disilicate prosthetic crowns were randomly assigned to one of the two decontamination methods. Clinical parameters, including probing pocket depth (PPD), bleeding on probing (BOP), and plaque index (PI), were evaluated at baseline (T0), six months (T1), and twelve months (T2). Statistical analysis was performed using Friedman’s test for repeated measures, followed by Dunn’s post hoc test. Subgroup analysis was conducted based on the prosthetic material. Results: Both treatment modalities led to statistically significant reductions in clinical parameters over 12 months. In the erythritol group, PPD decreased by 21.62%, BOP by 86.62%, and PI by 90.74%. In the ultrasonic group, PPD decreased by 14.86%, BOP by 78.69%, and PI by 64.86% (*p* < 0.05 for all). No statistically significant differences were observed between groups (*p* > 0.05). Subgroup analysis revealed similar clinical improvements across all crown materials, suggesting that treatment efficacy was not influenced by the type of prosthetic material. Conclusions: Both erythritol-based air polishing and ultrasonic instrumentation with PEEK inserts are effective and comparable in the maintenance of peri-implant health. As treatment outcomes were independent of crown composition, the choice between modalities should be tailored to patient-specific needs and clinical conditions. Future studies with a longer follow-up are recommended to evaluate the long-term impact on peri-implant tissue stability and to explore the role of prosthetic materials more comprehensively.

## 1. Introduction

Dental implants are a common therapeutic solution for replacing missing teeth in various clinical situations. However, one of the most common complications that can compromise implant success is peri-implant inflammation, which affects the surrounding hard and soft tissues. These inflammatory conditions are classified as mucositis and peri-implantitis [1,2,3].

Peri-implant mucositis is characterized by inflammation of the peri-implant soft tissues caused by the presence of bacteria, with clinical signs such as redness, swelling, and bleeding on probing (BOP). Although reversible, its resolution may require a longer time compared to gingivitis and is strongly dependent on both professional intervention and patient adherence. Mucositis should be considered a critical therapeutic window: if not properly managed, it significantly increases the risk of progression to peri-implantitis, especially in the absence of supportive care programs [4].

Peri-implantitis, on the other hand, is a progressive and irreversible condition involving both soft and hard tissues that can lead to bone loss, reduced osseointegration, peri-implant pocket formation, and, in some cases, suppuration [5,6]. As recently emphasized, peri-implantitis lesions are histologically more extensive than periodontal ones and exhibit a greater density of inflammatory infiltrates and vascular structures. In addition, the unique anatomy of peri-implant tissues—characterized by vertically oriented collagen fibers and reduced connective attachment—results in greater probing depth even in healthy sites, highlighting the importance of standardized diagnostic parameters and early intervention [4].

In addition to inflammatory complications, implant failure can result from excessive implant loading, material or technical defects, poor bone quality, and systemic conditions or pharmacological therapies that inhibit bone remodeling [7,8,9]. Prevention of peri-implant disease requires a structured approach that includes individualized risk assessment, minimization of risk factors, maintenance of optimal tissue conditions, and selection of the most appropriate implant type [3,10].

Preventing inflammation around dental implants is critical to ensuring their longevity and functionality, as well as maintaining the overall health of the patient [5]. Studies emphasize the importance of targeted preventive strategies, including proper oral hygiene, regular professional check-ups, and risk factor management. Daily oral hygiene, supported by manual or electric toothbrushes, interdental brushes, and special chlorhexidine-containing mouthwashes, helps prevent plaque accumulation and subsequent mucositis and peri-implantitis. Professional maintenance, including removal of bacterial debris and monitoring of clinical parameters such as BOP and pocket depth, further reduces the risk of disease progression [10,11,12,13,14,15]. This is consistent with findings from a recent systematic review, which highlighted that regular supportive implant care, including mechanical debridement with either ultrasonic or air polishing systems, significantly reduces the risk of peri-implant diseases. The review also stressed that patient compliance and individualized maintenance intervals are essential to long-term implant success [16].

An important aspect of prevention is the quality of materials and prosthetic design. Implants with surfaces treated to improve osseointegration and minimize bacterial colonization, together with well-designed prostheses, reduce the risk of inflammation [17]. Management of risk factors such as smoking, uncontrolled diabetes, and biomechanical stress is also critical as mitigating these factors has been shown to improve implant success rates [17,18,19]. Recent consensus statements, such as the S3-level clinical practice guideline published by the European Federation of Periodontology, strongly support the implementation of personalized preventive strategies throughout all phases of implant therapy—from treatment planning to prosthetic rehabilitation and maintenance. This guideline emphasizes the importance of risk factor control (e.g., smoking, diabetes, periodontitis) and biofilm management to prevent the onset and recurrence of peri-implant diseases, particularly highlighting the role of professional supportive care (SPIC) and early interventions for peri-implant mucositis as primary prevention of peri-implantitis [11].

Among biofilm control methods, air polishing with erythritol-based powders and ultrasonic decontamination with polyetheretherketone (PEEK) inserts have gained attention for their minimally invasive nature and clinical efficacy [20,21]. Erythritol, a biocompatible polyol with antibacterial properties, effectively disrupts biofilm while preserving PEEK implant soft tissue and prosthetic materials [22]. Clinical studies have demonstrated its efficacy in reducing PI, BOP, and PPD, with superior biofilm removal compared to conventional debridement and lower rates of gingival irritation. Its fine particle size enhances the removal of bacterial biofilm in peri-implant sulci while minimizing the risk of abrasion of implant surfaces [22,23,24].

Ultrasonic instruments with PEEK inserts provide an alternative approach to peri-implant maintenance, offering biocompatibility, mechanical efficiency, and minimal surface abrasiveness. PEEK, which is widely used in biomedical applications, has excellent mechanical stability and is gentle on both soft and hard tissue. Its non-metallic composition can help control peri-implant inflammation while maintaining the integrity of implant structures [25,26,27]. Studies highlight its efficacy in biofilm removal with minimal surface modification, reinforcing its clinical relevance [28,29]. Both erythritol air polishing and ultrasonic decontamination with PEEK inserts have been shown to be effective in maintaining peri-implant health. A randomized controlled trial found comparable reductions in PPD and BOP at 12 months, confirming their role as effective non-invasive maintenance approaches [23].

This study primarily aimed to evaluate the clinical effectiveness of two non-invasive professional hygiene protocols—erythritol-based air polishing and ultrasonic instrumentation with PEEK inserts—in maintaining peri-implant soft tissue health. A randomized clinical trial was conducted to assess their ability to reduce probing pocket depth (PPD), bleeding on probing (BOP), and plaque index (PI) over a 12-month follow-up period. The null hypothesis of the study was that there would be no significant difference between the two methods in improving peri-implant health parameters. A secondary objective was to determine whether the clinical effectiveness of these two protocols was influenced by the type of restorative material used for the implant-supported crowns—feldspathic ceramic, zirconia, or lithium disilicate. The goal was not to compare the materials themselves but to assess whether both methods performed equally well regardless of crown composition.

This randomized clinical trial contributes new evidence by combining a 12-month clinical follow-up with a subgroup analysis based on different prosthetic materials—an approach that remains underexplored in the current literature. To the best of our knowledge, few studies have integrated both aspects into a single design, making this study particularly relevant to the ongoing development of individualized peri-implant maintenance protocols.

## 2. Materials and Methods

This study was designed as a single-center randomized clinical trial and was conducted in accordance with ethical and regulatory standards. The research protocol was thoroughly reviewed and approved by the Unit Internal Review Board (approval number: 2023-0125). Furthermore, the trial was officially registered on ClinicalTrials.gov under the identifier NCT06496594, promoting transparency and accessibility of study details for researchers, clinicians, and stakeholders.

### 2.1. Participants

The research was conducted at the Unit of Dental Hygiene, Section of Dentistry, Department of Clinical, Surgical, Diagnostic, and Pediatric Sciences of the University of Pavia, Pavia, Italy. The study adhered to rigorous methodological standards to ensure the reliability and validity of the findings, with all procedures performed in accordance with ethical guidelines and institutional regulations.

The study population comprised patients presenting with at least one dental implant, with prosthetic crowns made of feldspathic ceramic, zirconia, or lithium disilicate. Participants were aged between 18 and 70 years and demonstrated good compliance with oral hygiene and follow-up visits. Prior to enrollment, all participants provided written informed consent after receiving a detailed explanation of the study’s objectives, procedures, and potential risks and benefits. Efforts were made to ensure that patients fully understood their involvement, reinforcing the ethical commitment to patient autonomy and well-being.

A set of exclusion criteria was established to ensure patient safety and the integrity of the study. Individuals with cardiac pacemakers were excluded due to potential interference with certain electronic equipment used during the interventions. Patients with neurological or psychological disorders, such as epilepsy, were also excluded, as these conditions could influence treatment response or pose risks during clinical procedures. Additionally, pregnant women were not included in the study, given that hormonal fluctuations and physiological changes associated with pregnancy could impact the clinical indices assessed. These exclusion criteria were carefully selected to minimize confounding variables and uphold the safety of all participants throughout the study period.

### 2.2. Intervention and Outcomes

At baseline (T0), after obtaining informed consent, patients underwent a comprehensive clinical periodontal examination. A calibrated operator assessed the following clinical indices using a UNC 12 plastic periodontal probe (Hu-Friedy, Chicago, IL, USA): Probing Pocket Depth (PPD), defined as the measurement in millimeters of the peri-implant sulcus, recorded at four sites around the gingival margin (vestibular, palatal/lingual, mesial, and distal) [30]; Bleeding on Probing (BoP), expressed as the percentage of sites exhibiting bleeding following periodontal probing [31]; and Plaque Index (PI), representing the percentage of tooth surfaces with visible plaque accumulation [32]. Periodontal probing was performed at four sites per implant (mesial, distal, buccal, and lingual), following previously validated protocols; this approach was selected to ensure consistency and reduce variability while still allowing reliable assessment of peri-implant health [30,33,34].

Following the initial assessment, patients were randomly assigned to one of the two treatment groups. In Group EAP, professional oral hygiene was performed using erythritol powder (AIRFLOW^®^ PLUS powder, Electro Medical Systems, Nyon, Switzerland), while in UPI, a piezoelectric handpiece equipped with a polyetheretherketone (PEEK)-coated plastic tip (Mini Piezon with PI, Electro Medical Systems, Nyon, Switzerland) was used; the patients were further subdivided into subgroups based on the material of the implant crown (EAP-CER/UPI-CER: feldspatic ceramic; EAP-ZIR/UPI-ZIR: zirconia; EAP-DISIL/UPI-DISIL: lithium disilicate). This subdivision was made because feldspathic ceramic, zirconia, and lithium disilicate are among the most commonly used materials for implant-supported crowns. Additionally, it aimed to assess whether clinical outcomes varied depending on the prosthetic material, providing insights into potential differences in treatment response.

Both interventions were standardized, with each implant receiving a total treatment duration of 30 s, allocating 5 s per site.

The professional oral hygiene sessions and the collection of the periodontal indexes were conducted after 6 months (T1) and after 12 months (T2).

### 2.3. Randomization

Participants were randomly assigned to one of the two treatment groups using a computer-generated randomization sequence to ensure an unbiased allocation process. A simple randomization method was employed, utilizing a random number generator to assign each enrolled patient to either the erythritol-based air-polishing group (Group EAP) or the ultrasonic instrumentation with PEEK inserts group (Group UPI). The randomization was performed using a 1:1 allocation ratio to ensure equal distribution across the two treatment arms. An independent researcher, who was not involved in the clinical procedures or outcome assessments, generated the sequence and managed the allocation process. The treatment assignment was concealed using sequentially numbered, opaque, sealed envelopes to prevent selection bias. Upon patient enrollment, a clinician opened the corresponding envelope to determine the assigned intervention. This approach ensured that the allocation process remained unpredictable and that all participants had an equal probability of receiving either treatment.

### 2.4. Sample Size

The sample size of the study was calculated by choosing the “Plaque Index” as the primary outcome. The calculation was based on the results of a previous study [30], setting alpha = 0.05 and power = 85% and hypothesizing an expected mean of 0.41, an expected mean difference of 0.30, and a standard deviation of 0.55. Therefore, 60 patients per group were required for the study.

### 2.5. Statistical Analysis

The data analysis has been carried out with the R software (R version 3.1.3, R Development Core Team, R Foundation for Statistical Computing, Wien, Austria) by calculating the descriptive statistics for each variable, which included mean, standard deviation, median, and minimum and maximum values, measured for each group.

For all tested parameters, the normality of the distributions was evaluated by the Shapiro–Wilk test. Due to the non-normal distribution of the data, a non-parametric test was applied (Friedman test), followed by post hoc testing (Dunn’s multiple comparison test). For all tests, the significance was set for *p* < 0.05.

## 3. Results

**Figure 1:**
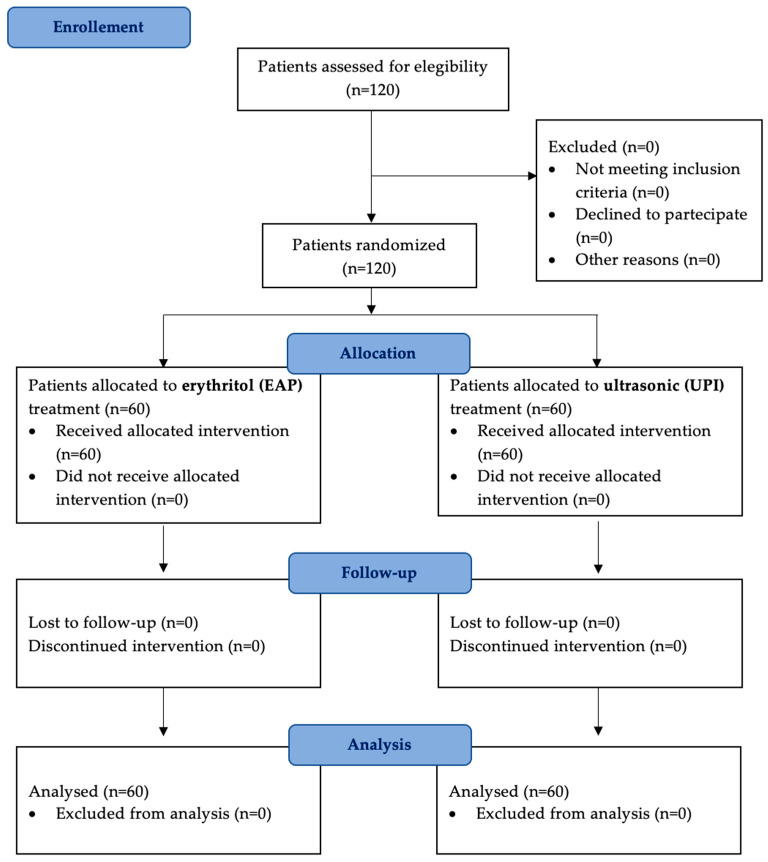
A flow diagram illustrating patient selection, randomization, and follow-up. A total of 120 patients were enrolled and randomly assigned to two treatment groups: erythritol-based air-polishing (Group EAP) and ultrasonic instrumentation with PEEK inserts (Group UPI). Clinical parameters (PPD, BOP, PI) were assessed at baseline (T0), six months (T1), and twelve months (T2). No patients were lost to follow-up.

A total of 120 patients fulfilled the inclusion criteria, provided informed consent, and underwent the assigned treatments. The study started in March 2023 and concluded in April 2024. All participants who were initially enrolled completed the study and were included in the final analysis, with no exclusions (Figure 1).

The study included a total of 120 patients, with a nearly equal distribution of males (51.67%) and females (48.33%). The mean age of the participants was 50.29 ± 10.76 years for males and 49.59 ± 10.54 years for females, indicating a homogeneous age distribution. When analyzed by treatment group, the erythritol-based air-polishing group (EAP) comprised 29 males (24.17%) and 31 females (25.83%), with mean ages of 49.24 ± 10.38 and 48.52 ± 11.98 years, respectively. The ultrasonic instrumentation with PEEK inserts group (UPI) included 33 males (27.50%) and 27 females (22.50%), with slightly higher mean ages of 51.21 ± 11.15 and 50.81 ± 8.66 years. The balanced demographic distribution between groups ensures the comparability of treatment outcomes, minimizing potential biases related to age or sex (Table 1).

Participants were further classified based on the type of prosthetic material used for their implant-supported crowns, forming six subgroups. The feldspathic ceramic subgroups (EAP-CER/UPI-CER) included 20 patients for group (EAP/UPI), with 9 males and 11 females in the erythritol-based air-polishing group (EAP-CER) and 12 males and 8 females in the ultrasonic instrumentation with PEEK inserts group (UPI-CER). The zirconia subgroups (EAP-ZIR/UPI-ZIR) comprised 40 participants, with a similar gender distribution across treatment arms: 20 patients for group (EAP/UPI), with 11 males and 9 females in the erythritol-based air-polishing group (EAP-ZIR) and with 11 males and 9 females in the ultrasonic instrumentation with PEEK inserts group (UPI-ZIR). The lithium disilicate subgroups (EAP-DISIL/UPI-DISIL) had 40 patients, maintaining a balanced allocation between males and females: 20 patients for group (EAP/UPI), with 9 males and 11 females in the erythritol-based air-polishing group (EAP-DISIL) and 9 males and 11 females in the ultrasonic instrumentation with PEEK inserts group (UPI-DISIL). Mean ages were comparable across all subgroups, confirming that prosthetic material distribution did not introduce significant demographic differences between treatment groups (Table 2).

**Figure 2:**
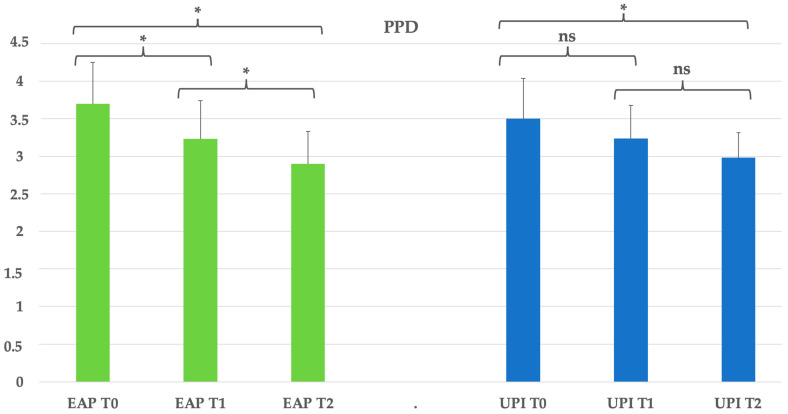
Group EAP (erythritol-based air-polishing) and Group UPI (ultrasonic instrumentation with PEEK inserts). The graphic in the figure shows intragroup differences: the asterisk indicates a statistically significant difference, while “ns” indicates no statistically significant difference; no intergroup difference was detected (*p* > 0.05).

Regarding the periodontal indexes, the reduction in probing pocket depth was significant in both treatment groups, from T0 to T2 (*p* < 0.05). The erythritol-based air-polishing group (EAP) showed a progressive decrease from 3.7 mm at T0 to 3.23 mm at T1, reaching 2.9 mm at T2, corresponding to a 21.6% reduction. The ultrasonic instrumentation with PEEK inserts group (UPI) exhibited a similar pattern, decreasing from 3.5 mm at T0 to 3.24 mm at T1 and 2.98 mm at T2, resulting in a 14.9% reduction. See Table 3 and Figure 2.

**Figure 3:**
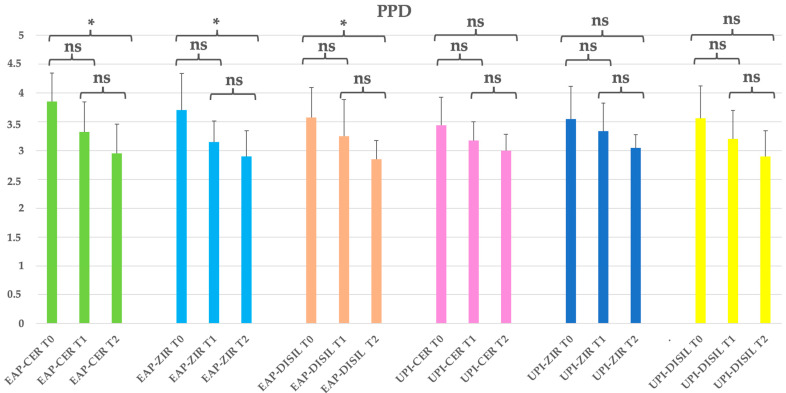
EAP-CER (erythritol-based air-polishing group with feldspathic ceramic crown), EAP-ZIR (erythritol-based air-polishing group with zirconia crown), EAP-DISIL (erythritol-based air-polishing group with lithium disilicate crown), UPI-CER (ultrasonic instrumentation with PEEK inserts group with feldspathic ceramic crown), UPI-ZIR (ultrasonic instrumentation with PEEK inserts group with zirconia crown), UPI-DISIL (ultrasonic instrumentation with PEEK inserts group with lithium disilicate crown). The graphic in the figure shows intragroup differences: the asterisk indicates a statistically significant difference, while “ns” indicates no statistically significant difference; no intergroup difference was detected (*p* > 0.05).

A reduction in probing pocket depth (PPD) was observed over time across all EAP subgroups (erythritol-based air polishing), regardless of the prosthetic material used. A statistically significant decrease was found between baseline (T0) and the final follow-up (T2) (*p* < 0.05). The highest baseline PPD values were recorded in the EAP-CER and EAP-ZIR subgroups, with mean values of 3.85 mm and 3.70 mm, respectively. By the end of the study, all EAP subgroups showed mean PPD values below 3 mm, corresponding to reductions of 23.4% (CER), 21.6% (ZIR), and 20.2% (DISIL). In the EAP group, the reduction in PPD was significant for all materials. In contrast, the UPI subgroups did not show statistically significant reductions over time (*p* > 0.05), although modest decreases were observed: 12.8% (CER), 14.1% (ZIR), and 18.5% (DISIL). Additionally, no statistically significant differences were found between prosthetic materials within each group at the final time point (*p* > 0.05). Table 4; Figure 3.

**Figure 4:**
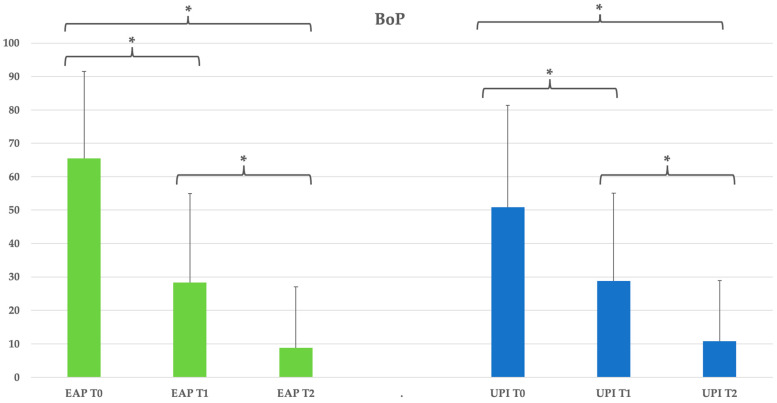
Group EAP (erythritol-based air-polishing) and Group UPI (ultrasonic instrumentation with PEEK inserts). The graphic in the figure shows intragroup differences: the asterisk indicates a statistically significant difference, while “ns” indicates no statistically significant difference; no intergroup difference was detected (*p* > 0.05).

The bleeding on probing (BOP) significantly improved from T0 to both T1 and T2 in the erythritol group (*p* < 0.05), decreasing from 65.42% at T0 to 28.33% at T1 and further to 8.75% at T2, corresponding to an 86.6% reduction. A similar trend was observed in the ultrasonic group, with BOP reducing from 50.83% at T0 to 28.75% at T1 and 10.83% at T2, representing a 78.8% reduction, also with statistical significance (*p* < 0.05). No significant intergroup differences were detected at any follow-up point (*p* > 0.05). See Table 5 and Figure 4.

**Figure 5:**
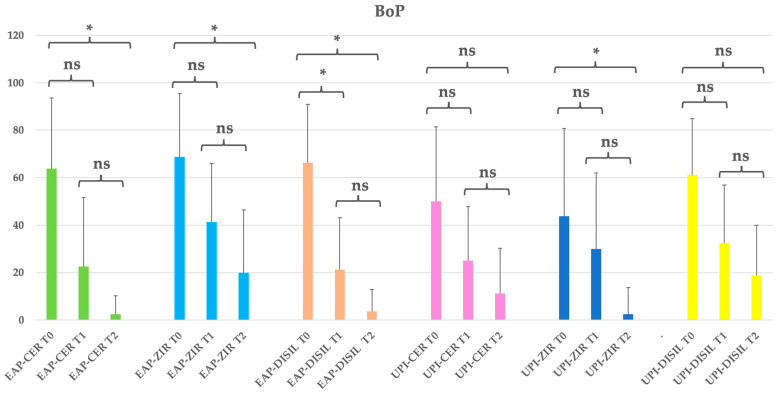
EAP-CER (erythritol-based air-polishing group with feldspathic ceramic crown), EAP-ZIR (erythritol-based air-polishing group with zirconia crown), EAP-DISIL (erythritol-based air-polishing group with lithium disilicate crown), UPI-CER (ultrasonic instrumentation with PEEK inserts group with feldspathic ceramic crown), UPI-ZIR (ultrasonic instrumentation with PEEK inserts group with zirconia crown), UPI-DISIL (ultrasonic instrumentation with PEEK inserts group with lithium disilicate crown). The graphic in the figure shows intragroup differences: the asterisk indicates a statistically significant difference, while “ns” indicates no statistically significant difference; no intergroup difference was detected (*p* > 0.05).

All subgroups exhibited a progressive reduction in bleeding on probing (BoP) over time, with statistically significant differences observed between T0 and T2 (*p* < 0.05). The most substantial reductions were noted in the EAP-CER and EAP-DISIL subgroups, which showed decreases of 96.1% and 94.3%, respectively. At baseline, BoP values ranged from 43.75% to 68.75% across groups. By the end of the study (T2), nearly all subgroups showed mean BoP values below 10%, indicating effective inflammation control. Notably, the EAP-CER and EAP-DISIL subgroups demonstrated reductions of 2.5% and 3.75%, respectively. Although the UPI subgroups also demonstrated reductions—77.5% (CER), 94.3% (ZIR), and 69.4% (DISIL)—the changes were generally less pronounced and did not consistently reach statistical significance. These findings highlight the overall effectiveness of both treatment modalities—erythritol-based air polishing and ultrasonic instrumentation—in reducing peri-implant mucosal inflammation, with a trend toward greater efficacy observed in the EAP-treated subgroups. See Table 6 and Figure 5.

**Figure 6:**
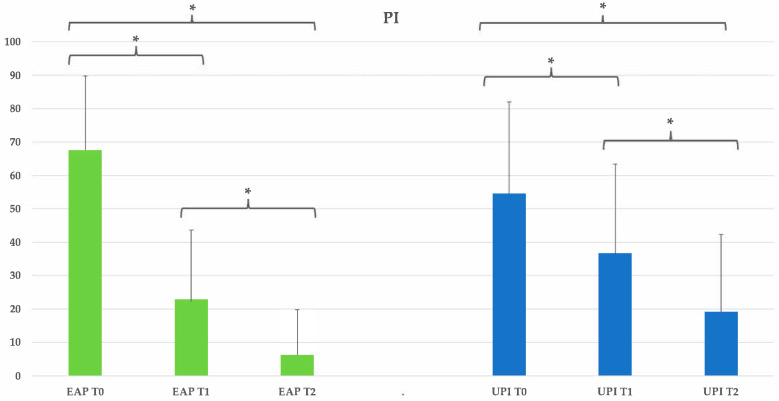
Group EAP (erythritol-based air-polishing) and Group UPI (ultrasonic instrumentation with PEEK inserts). The graphic in the figure shows intragroup differences: the asterisk indicates a statistically significant difference; no intergroup difference was detected (*p* > 0.05).

Plaque accumulation significantly decreased in both groups over time. In the erythritol group, PI reduced from 67.5% at T0 to 22.92% at T1 and 6.25% at T2, corresponding to a 90.7% reduction (*p* < 0.05). The ultrasonic group showed a similar trend, with PI decreasing from 54.58% at T0 to 36.67% at T1 and 19.17% at T2, resulting in a 64.9% reduction (*p* < 0.05). No statistically significant differences between the two treatment modalities were found at any time point (*p* > 0.05). See Table 7 and Figure 6.

**Figure 7:**
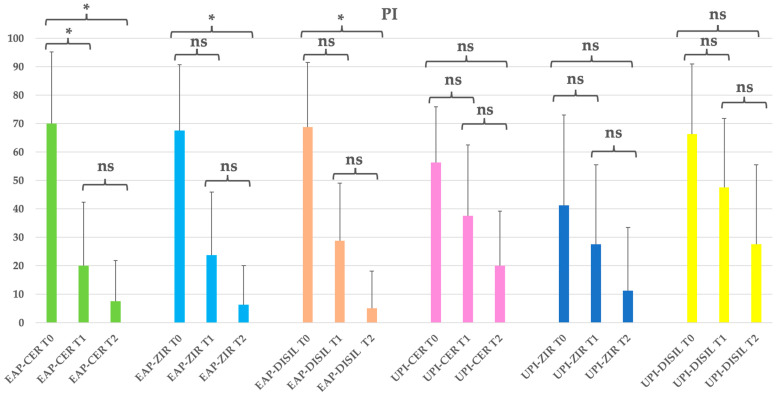
EAP-CER (erythritol-based air-polishing group with feldspathic ceramic crown), EAP-ZIR (erythritol-based air-polishing group with zirconia crown), EAP-DISIL (erythritol-based air-polishing group with lithium disilicate crown), UPI-CER (ultrasonic instrumentation with PEEK inserts group with feldspathic ceramic crown), UPI-ZIR (ultrasonic instrumentation with PEEK inserts group with zirconia crown), UPI-DISIL (ultrasonic instrumentation with PEEK inserts group with lithium disilicate crown). The graphic in the figure shows intragroup differences: the asterisk indicates a statistically significant difference, while “ns” indicates no statistically significant difference; no intergroup difference was detected (*p* > 0.05).

A significant reduction in plaque index (PI) was observed across all subgroups from baseline to the final follow-up (*p* < 0.05), with more pronounced decreases in the groups treated with erythritol-based air polishing (EAP). At baseline, PI values ranged from 41.25% to 70.00%. By T2, the EAP subgroups showed the lowest mean plaque levels, with values dropping to 7.5%, 6.25%, and 5.00% in the EAP-CER, EAP-ZIR, and EAP-DISIL subgroups, respectively, corresponding to reductions of 89.3%, 90.7%, and 92.7%. The UPI subgroups also demonstrated reductions over time, though to a lesser extent and without reaching statistical significance in most comparisons: 64.4% (UPI-CER), 72.7% (UPI-ZIR), and 58.5% (UPI-DISIL). Despite the observed reductions, no statistically significant differences were found between the different prosthetic materials at the end of the study (*p* > 0.05), suggesting that the method of decontamination has a greater impact on plaque control than the type of implant crown material. See Table 8 and Figure 7.

## 4. Discussion

This single-center randomized clinical trial provides valuable insights into the efficacy of erythritol-based air-polishing and ultrasonic decontamination with PEEK inserts in maintaining peri-implant health. The study evaluated three key clinical parameters—probing pocket depth (PPD), bleeding on probing (BOP), and plaque index (PI). The null hypothesis of the study was accepted as it was demonstrated that no statistically significant differences between both treatment modalities occurred, even though a significant improvement over time was observed for the intragroup analysis. These findings highlight the importance of mechanical biofilm disruption in peri-implant maintenance, reinforcing the notion that the effectiveness of professional decontamination protocols relies more on the consistent removal of bacterial deposits than on the specific type of mechanical intervention employed [13].

The progressive reduction in PPD observed in both groups (EAP/UPI) confirms the efficacy of biofilm control strategies in peri-implant maintenance. The slight difference between groups, with the erythritol group showing a marginally greater reduction, did not reach statistical significance, supporting the hypothesis that both air-polishing and ultrasonic instrumentation effectively contribute to peri-implant tissue health and stability. The analysis of subgroups based on prosthetic crown materials confirmed a consistent reduction in probing pocket depth across all groups, suggesting that the effectiveness of both decontamination methods is not influenced by the type of prosthetic material. These results are in line with previous research demonstrating that non-surgical interventions, including air-polishing and ultrasonic debridement, are essential in preventing disease progression and promoting tissue healing [35,36,37]. Additionally, the consistent improvement in PPD across all follow-up periods suggests that regular professional maintenance sessions play a pivotal role in sustaining peri-implant health over time [38]. Given that pocket depth reduction is a key indicator of successful biofilm control and inflammation resolution [11], our findings support the integration of both techniques into routine peri-implant maintenance protocols. The improvements in BOP further reinforce the role of both treatment approaches in reducing peri-implant soft tissue inflammation. The more rapid initial reduction observed in the erythritol group at T1 may indicate a short-term anti-inflammatory effect, potentially linked to the physical properties of erythritol powder, which has been previously described as having antibacterial and biofilm-disrupting capabilities. However, by T2, both modalities achieved comparable levels of improvement, confirming that both methods are equally effective in long-term inflammation control. This aligns with previous findings indicating that BOP reduction is a key parameter in peri-implant health improvement as it reflects a decrease in microbial-induced tissue inflammation [39]. Similarly, when evaluating bleeding on probing across subgroups, all prosthetic materials demonstrated a clear improvement over time. While some variations were observed in the initial response to treatment, the long-term reduction in inflammation remained comparable, highlighting the reliability of both approaches in maintaining peri-implant soft tissue health. The fact that no significant intergroup differences were observed suggests that mechanical disruption of the biofilm, rather than the specific instrument used, is the determining factor in reducing inflammation. These findings further validate the role of professional maintenance therapies in preventing the progression of peri-implant mucositis to peri-implantitis, which remains a major concern in implant dentistry [40].

Plaque accumulation is one of the primary etiological factors in peri-implant disease, making its control a fundamental aspect of long-term implant maintenance. The significant reduction in PI observed in both groups (EAP/UPI) highlights the effectiveness of both approaches in controlling biofilm accumulation. Previous studies have also demonstrated the efficacy of air-abrasive debridement with glycine powder as a viable alternative for peri-implant maintenance, showing comparable results to mechanical debridement and chlorhexidine administration over a six-month period [41]. Although the erythritol group exhibited slightly lower PI values at each time point, patient compliance and comfort may have played a role in this trend. Plaque control followed the same pattern across different prosthetic materials, with both treatment modalities contributing effectively to biofilm removal. The similarities between subgroups further suggest that successful plaque management is primarily linked to the mechanical decontamination approach rather than the specific prosthetic material used. Air-polishing with erythritol has been reported to be more comfortable and less invasive compared to ultrasonic instrumentation, which may contribute to better patient adherence to maintenance recommendations [42]. Previous studies have suggested that the perception of comfort during professional hygiene procedures can influence patient motivation and compliance, which, in turn, affects the long-term success of implant maintenance strategies [43,44].

However, individual home oral hygiene routines were not standardized or monitored during the follow-up. Patient compliance was assessed through adherence to scheduled recall appointments, rather than through direct evaluation of home-care behavior. Further research should explore whether the perceived comfort associated with erythritol air-polishing translates into improved long-term clinical outcomes, particularly in patient populations prone to plaque accumulation or reduced adherence to oral hygiene protocols. Alongside these clinical and patient-related aspects, the study also considered whether the type of prosthetic crown material might influence the effectiveness of the maintenance protocols. This study was not designed to compare the clinical performance of different prosthetic materials but rather to assess whether the choice of material affected the clinical effectiveness of the maintenance protocols. The descriptive subgroup analysis showed similar trends in clinical improvement across the different prosthetic materials, suggesting a potential independence of treatment efficacy from the crown composition. However, no direct statistical comparisons were performed between materials, and thus, this observation should be interpreted as exploratory. Further studies specifically designed to assess the impact of restorative materials on maintenance outcomes are warranted.

From a clinical perspective, both erythritol-based air-polishing and ultrasonic instrumentation with PEEK inserts appear to be effective, minimally invasive, and safe for peri-implant maintenance. The low-abrasiveness properties of erythritol-based air-polishing make it a particularly attractive option for patients with thin peri-implant soft tissues or esthetic zone implants, where excessive mechanical trauma should be avoided [22,23,45]. On the other hand, ultrasonic decontamination with PEEK inserts provides an effective means of mechanical biofilm removal, making it particularly suitable in cases where a more intensive decontamination approach is required, such as in patients with higher plaque accumulation, deeper peri-implant pockets, or a history of peri-implant inflammation [21,22,46]. These complementary characteristics may help guide clinical decision-making, allowing clinicians to tailor maintenance strategies based on tissue type, plaque levels, or patient sensitivity, rather than considering one method as universally superior.

In interpreting these results, several methodological limitations of the present trial must be acknowledged. First, as a single-center study, the generalizability of the findings may be constrained by the specific clinical setting and patient population. Second, the absence of operator blinding may have introduced performance bias, even though outcome measures were objective and standardized. Third, the lack of microbiological or biochemical assessments limited the understanding of the biological mechanisms underlying treatment efficacy. Additionally, the study did not exclude patients with systemic conditions such as smoking or diabetes, which are known to affect peri-implant tissue health. While this enhances external validity, it may also introduce confounding effects. Moreover, data on the timing of implant and prosthetic crown placement were not collected. Although all patients were enrolled in regular maintenance programs, implant age might influence tissue stability and should be considered in future studies. Home oral hygiene routines were not standardized or monitored during the follow-up period. Patient compliance was assessed based on attendance at scheduled maintenance visits rather than direct evaluation of at-home hygiene behavior. The lack of patient-reported outcomes, such as perceived comfort or satisfaction, also represents a limitation, as these aspects are increasingly relevant in guiding adherence and long-term success. Expanding future research to include these dimensions would enhance the clinical relevance and patient-centered applicability of peri-implant maintenance protocols.

Future research should aim to incorporate larger or more diverse populations and extended follow-up periods. Additionally, recently introduced tools—such as microbiological assessments [47], biomarker analyses [48], integrated applications [49], and AI-based software [50]—would be useful in better understanding the biological mechanisms driving peri-implant disease progression and treatment response. The combined effect of different adjunctive treatments, such as non-pharmacological decontamination [51], ozone application [52], and photobiomodulation [53], should be considered in future reports. Furthermore, studies comparing these techniques in high-risk populations (e.g., smokers, diabetic patients, or individuals with a history of peri-implant disease) could provide valuable insights into optimizing maintenance protocols for different patient profiles. Understanding how these approaches perform in more challenging clinical scenarios would help refine treatment selection criteria and contribute to the development of evidence-based guidelines for peri-implant maintenance.

## 5. Conclusions

In conclusion, both erythritol-based air polishing and ultrasonic instrumentation with PEEK inserts proved to be effective and safe methods for peri-implant maintenance, with no statistically significant differences between the two protocols. At 12 months, the erythritol group showed an 86.6% reduction in BOP and a 90.7% reduction in PI, while the ultrasonic group achieved reductions of 78.7% and 64.9%, respectively. These findings confirm the relevance of mechanical biofilm disruption and support the integration of both approaches into routine maintenance strategies. From a clinical perspective, erythritol-based air polishing may be preferable in patients with thin soft tissues, esthetic zone restorations, or sensitivity to mechanical vibration as it is minimally invasive and well tolerated. Ultrasonic decontamination with PEEK inserts may be favored in cases with tenacious deposits, deeper pockets, or a history of peri-implant inflammation. Clinicians should also consider patient preferences, comfort perception, and cost-effectiveness when tailoring individualized maintenance protocols. Future research is encouraged to explore these modalities in diverse populations and to include patient-reported outcomes to guide truly personalized peri-implant care.

## Figures and Tables

**Table 1:** Cumulative demographic characteristics of patients in Group EAP (erythritol-based air-polishing) and Group UPI (ultrasonic instrumentation with PEEK inserts). The table presents the distribution of participants by sex and mean age and standard deviation (SD) for each treatment group.

Patients	Sex	n (%)	Mean Age (SD)
Total	Males	62 (51.67%)	50.29 (10.76)
	Females	58 (48.33%)	49.59 (10.54)
EAP	Males	29 (24.17%)	49.24 (10.38)
	Females	31 (25.83%)	48.52 (11.98)
UPI	Males	33 (27.50%)	51.21 (11.15)
	Females	27 (22.50%)	50.81 (8.66)

**Table 2:** Demographic distribution of participants based on the prosthetic crown material used (feldspathic ceramic, zirconia, lithium disilicate) in both treatment groups. EAP-CER (erythritol-based air-polishing group with feldspathic ceramic crown), EAP-ZIR (erythritol-based air-polishing group with zirconia crown), EAP-DISIL (erythritol-based air-polishing group with lithium disilicate crown), UPI-CER (ultrasonic instrumentation with PEEK inserts group with feldspathic ceramic crown), UPI-ZIR (ultrasonic instrumentation with PEEK inserts group with zirconia crown), UPI-DISIL (ultrasonic instrumentation with PEEK inserts group with lithium disilicate crown). The table presents the number of males and females in each subgroup, along with their mean age (±standard deviation), ensuring comparability across different prosthetic materials.

Patients	Sex	n (%)	Mean Age (SD)
Total	Males	62 (51.67%)	50.29 (10.76)
	Females	58 (48.33%)	49.59 (10.54)
EAP-CER	Males	9 (7.50%)	48.00 (9.99)
	Females	11 (9.17%)	45.45 (12.23)
EAP-ZIR	Males	11 (9.17%)	52.82 (8.96)
	Females	9 (7.50%)	52.56 (10.48)
EAP-DISIL	Males	9 (7.50%)	46.11 (10.49)
	Females	11 (9.17%)	48.27 (11.38)
UPI-CER	Males	12 (10.00%)	51.33 (12.11)
	Females	8 (6.67%)	51.63 (9.81)
UPI-ZIR	Males	11 (9.17%)	50.64 (12.65)
	Females	9 (7.50%)	51.89 (5.78)
UPI-DISIL	Males	9 (7.50%)	52.00 (7.06)
	Females	11 (9.17%)	49.18 (8.73)

**Table 3:** Clinical parameters overview and statistical significance (PPD: probing pocket depth). Group EAP (erythritol-based air-polishing) and Group UPI (ultrasonic instrumentation with PEEK inserts). The table presents the mean, standard deviation (St Dev), minimum (Min), median, and maximum (Max) values for each clinical parameter assessed at different time points (T0, T1, T2) in both treatment groups.

Group	Time	Mean	St Dev	Min	Median	Max
EAP	T0	3.70	0.55	3.00	4.00	5.00
	T1	3.23	0.51	2.00	3.00	4.50
	T2	2.90	0.43	2.00	3.00	4.00
UPI	T0	3.50	0.54	3.00	3.25	4.50
	T1	3.24	0.44	2.50	3.00	4.50
	T2	2.98	0.33	2.00	3.00	4.00

**Table 4:** Clinical parameters overview and statistical significance (PPD: probing pocket depth) by prosthetic material subgroups. EAP-CER (erythritol-based air-polishing group with feldspathic ceramic crown), EAP-ZIR (erythritol-based air-polishing group with zirconia crown), EAP-DISIL (erythritol-based air-polishing group with lithium disilicate crown), UPI-CER (ultrasonic instrumentation with PEEK inserts group with feldspathic ceramic crown), UPI-ZIR (ultrasonic instrumentation with PEEK inserts group with zirconia crown), UPI-DISIL (ultrasonic instrumentation with PEEK inserts group with lithium disilicate crown). The table presents the mean, standard deviation (St Dev), minimum (Min), median, and maximum (Max) values for each clinical parameter assessed at different time points (T0, T1, T2) in both treatment groups.

Group	Time	Mean	St Dev	Min	Median	Max
EAP-CER	T0	3.85	0.50	3.00	4.00	4.75
	T1	3.32	0.52	2.00	3.00	4.50
	T2	2.95	0.51	2.00	3.00	4.00
EAP-ZIR	T0	3.70	0.64	3.00	3.87	5.00
	T1	3.15	0.37	3.00	3.00	4.00
	T2	2.90	0.45	2.00	3.00	4.00
EAP-DISIL	T0	3.57	0.52	3.00	3.75	4.50
	T1	3.25	0.64	2.00	3.00	4.00
	T2	2.85	0.33	2.00	3.00	3.00
UPI-CER	T0	3.44	0.48	3.00	3.12	4.00
	T1	3.17	0.32	2.00	3.00	4.00
	T2	3.00	0.28	2.00	3.00	3.50
UPI-ZIR	T0	3.55	0.56	3.00	3.50	4.50
	T1	3.34	0.49	3.00	3.00	4.50
	T2	3.05	0.22	3.00	3.00	4.00
UPI-DISIL	T0	3.56	0.56	3.00	3.62	4.50
	T1	3.20	0.50	2.50	3.00	4.00
	T2	2.90	0.45	2.00	3.00	4.00

**Table 5:** Clinical parameters overview and statistical significance (BoP: bleeding on probing). Group EAP (erythritol-based air-polishing) and Group UPI (ultrasonic instrumentation with PEEK inserts). The table presents the mean, standard deviation (St Dev), minimum (Min), median, and maximum (Max) values for each clinical parameter assessed at different time points (T0, T1, T2) in both treatment groups.

Group	Time	Mean	St Dev	Min	Median	Max
EAP	T0	65.42	26.08	25.00	50.00	100.00
	T1	28.33	26.63	0.00	25.00	100.00
	T2	8.75	18.31	0.00	0.00	100.00
UPI	T0	50.83	30.52	0.00	50.00	100.00
	T1	28.75	26.37	0.00	25.00	100.00
	T2	10.83	18.04	0.00	0.00	50.00

**Table 6:** Clinical parameters overview and statistical significance (BoP: bleeding on probing) by prosthetic material subgroups. EAP-CER (erythritol-based air-polishing group with feldspathic ceramic crown), EAP-ZIR (erythritol-based air-polishing group with zirconia crown), EAP-DISIL (erythritol-based air-polishing group with lithium disilicate crown), UPI-CER (ultrasonic instrumentation with PEEK inserts group with feldspathic ceramic crown), UPI-ZIR (ultrasonic instrumentation with PEEK inserts group with zirconia crown), UPI-DISIL (ultrasonic instrumentation with PEEK inserts group with lithium disilicate crown). The table presents the mean, standard deviation (St Dev), minimum (Min), median, and maximum (Max) values for each clinical parameter assessed at different time points (T0, T1, T2) in both treatment groups.

Group	Time	Mean	St Dev	Min	Median	Max
EAP-CER	T0	63.75	29.77	25.00	50.00	100.00
	T1	22.50	29.13	0.00	0.00	75.00
	T2	2.50	7.69	0.00	0.00	25.00
EAP-ZIR	T0	68.75	26.75	25.00	25.00	100.00
	T1	41.25	24.70	0.00	0.00	100.00
	T2	20.00	26.40	0.00	0.00	100.00
EAP-DISIL	T0	66.25	24.70	25.00	25.00	100.00
	T1	21.25	21.88	0.00	0.00	50.00
	T2	3.75	9.16	0.00	0.00	25.00
UPI-CER	T0	50.00	31.41	0.00	50.00	100.00
	T1	25.00	22.94	0.00	25.00	50.00
	T2	11.25	18.98	0.00	0.00	50.00
UPI-ZIR	T0	43.75	37.06	0.00	50.00	100.00
	T1	30.00	32.04	0.00	25.00	100.00
	T2	2.50	11.18	0.00	0.00	50.00
UPI-DISIL	T0	61.25	23.61	0.00	50.00	100.00
	T1	32.50	24.47	0.00	50.00	50.00
	T2	18.75	21.27	0.00	12.50	50.00

**Table 7:** Clinical parameters overview and statistical significance (PI:plaque index). Group EAP (erythritol-based air-polishing) and Group UPI (ultrasonic instrumentation with PEEK inserts). The table presents the mean, standard deviation (St Dev), minimum (Min), median, and maximum (Max) values for each clinical parameter assessed at different time points (T0, T1, T2) in both treatment groups.

Group	Time	Mean	St Dev	Min	Median	Max
EAP	T0	67.50	22.22	25.00	50.00	100.00
	T1	22.92	20.73	0.00	25.00	50.00
	T2	6.25	13.52	0.00	0.00	50.00
UPI	T0	54.58	27.42	0.00	50.00	100.00
	T1	36.67	26.63	0.00	50.00	100.00
	T2	19.17	23.18	0.00	0.00	75.00

**Table 8:** Clinical parameters overview and statistical significance (PI: plaque index) by Prosthetic material Subgroups. EAP-CER (erythritol-based air-polishing group with feldspathic ceramic crown), EAP-ZIR (erythritol-based air-polishing group with zirconia crown), EAP-DISIL (erythritol-based air-polishing group with lithium disilicate crown), UPI-CER (ultrasonic instrumentation with PEEK inserts group with feldspathic ceramic crown), UPI-ZIR (ultrasonic instrumentation with PEEK inserts group with zirconia crown), UPI-DISIL (ultrasonic instrumentation with PEEK inserts group with lithium disilicate crown). The table presents the mean, standard deviation (St Dev), minimum (Min), median, and maximum (Max) values for each clinical parameter assessed at different time points (T0, T1, T2) in both treatment groups.

Group	Time	Mean	St Dev	Min	Median	Max
EAP-CER	T0	70.00	25.13	25.00	62.50	100.00
	T1	20.00	22.36	0.00	12.50	50.00
	T2	7.50	14.28	0.00	0.00	50.00
EAP-ZIR	T0	67.50	23.08	50.00	50.00	100.00
	T1	23.75	22.18	0.00	25.00	50.00
	T2	6.25	13.75	0.00	0.00	50.00
EAP-DISIL	T0	68.75	22.76	50.00	50.00	100.00
	T1	28.75	20.32	0.00	25.00	50.00
	T2	5.00	13.08	0.00	0.00	50.00
UPI-CER	T0	56.25	19.66	25.00	50.00	100.00
	T1	37.50	25.00	0.00	50.00	100.00
	T2	20.00	19.19	0.00	25.00	50.00
UPI-ZIR	T0	41.25	31.70	0.00	50.00	100.00
	T1	27.50	27.98	0.00	25.00	75.00
	T2	11.25	22.18	0.00	0.00	75.00
UPI-DISIL	T0	66.25	24.70	25.00	50.00	100.00
	T1	47.50	24.20	0.00	50.00	100.00
	T2	27.50	27.98	0.00	25.00	75.00

## Data Availability

The original contributions presented in this study are included in the article. Further inquiries can be directed to the corresponding authors.
